# Cerium Oxide and Chondroitin Sulfate Doped Polyurethane Scaffold to Bridge Tendons

**DOI:** 10.1021/acsami.3c06144

**Published:** 2023-05-23

**Authors:** Eleonora Bianchi, Marco Ruggeri, Barbara Vigani, Elena Del Favero, Caterina Ricci, Cinzia Boselli, Antonia Icaro Cornaglia, César Viseras, Silvia Rossi, Giuseppina Sandri

**Affiliations:** † Department of Drug Sciences, University of Pavia, Viale Taramelli 12, Pavia 27100, Italy; ‡ Department of Medical Biotechnology and Translational Medicine, University of Milan, LITA Viale Fratelli Cervi 93, Segrate 20090, Italy; § Department of Public Health, Experimental and Forensic Medicine, University of Pavia, via Forlanini 2, Pavia 27100 , Italy; ∥ Department of Pharmacy and Pharmaceutical Technology, Faculty of Pharmacy, University of Granada, Campus of Cartuja, Granada 18071, Spain

**Keywords:** electrospinning, tendon disorders, thermoplastic polyurethane, chondroitin sulfate, cerium oxide, mechanical properties

## Abstract

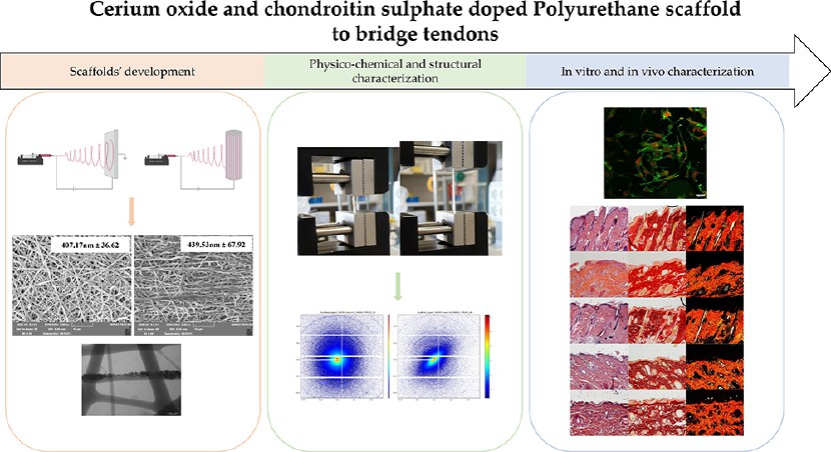

Tendon disorders are common medical conditions, which can be greatly debilitating as they are often accompanied by great pain and inflammation. The techniques used nowadays for the treatment of chronic tendon injuries often involve surgery. However, one critical aspect of this procedure involves the scar tissue, characterized by mechanical properties that vary from healthy tissue, rendering the tendons inclined to reinjury or rupture. Synthetic polymers, such as thermoplastic polyurethane, are of special interest in the tissue engineering field as they allow the production of scaffolds with controlled elastic and mechanical properties, which could guarantee an effective support during the new tissue formation. The aim of this work was the design and the development of tubular nanofibrous scaffolds based on thermoplastic polyurethane and enriched with cerium oxide nanoparticles and chondroitin sulfate. The scaffolds were characterized by remarkable mechanical properties, especially when tubular aligned, reaching values comparable to the ones of the native tendons. A weight loss test was performed, suggesting a degradation in prolonged times. In particular, the scaffolds maintained their morphology and also remarkable mechanical properties after 12 weeks of degradation. The scaffolds promoted the cell adhesion and proliferation, in particular when in aligned conformation. Finally, the systems in vivo did not cause any inflammatory effect, representing interesting platforms for the regeneration of injured tendons.

## 1 Introduction

Tendon disorders result from common health conditions, such as overuse injuries, ruptures, and also inflammatory and degenerative alterations. They can be greatly debilitating as they are often accompanied by great pain and inflammation. Moreover, they could be related to intertendinous degeneration, with consequent tissue rupture. More than 50% of all musculoskeletal injuries that are reported every year involve tendon and ligament injuries.^1,2^ This type of injuries appears principally in subjects who are physically active, independently of their age, but they are also found in the sedentary population as a result of intrinsic factors, including nutrition, aging, genetic diseases, and body weight.^3^

The techniques used nowadays for the treatment of chronic tendon injuries often involve surgery. However, one critical aspect of this procedure involves the scar tissue, characterized by different mechanical properties than healthy tissue. This renders the tendons prone to reinjury or rupture, leading to relapse.^4^ For this reason, new approaches need to be explored. Biopolymer-based scaffolds have been proposed in tissue engineering to replace and restore damaged tendon tissue because they possess the ability to mimic the structural, biochemical, and biomechanical functions of the extracellular matrix (ECM), consequently mimicking the native tissues. In particular, in orthopedic tissue regeneration, biodegradable polymeric implants based on poly-d-and poly-l-lactic acid, which represent the first generation of thermoplastic biodegradable polymers, have been researched as a substitute to traditional implants, avoiding the necessity of a second surgery to remove the implant.^5,6^ These materials are free from toxic and mutagenic effects, but they also have several issues, most importantly mechanical stiffness, unfavorable tissue responses, and foreign body reactions.^7,8^ On the other side, natural polymers, such as polysaccharides, are very similar in composition to the components of the native ECM, avoiding toxicity and immunological reactions, but they are also deficient in mechanical properties, which are fundamental to effectively mimic and support the tissue during the regeneration process and to induce the mechanotransduction of cell response, fundamental for the stimulation of specific stem cell differentiation.^9,10^ For these reasons, new synthetic polymers, such as thermoplastic polyurethane (TPU), are of special interest as they allow the production of scaffolds with controlled elastic and mechanical properties that could guarantee an effective support during the new tissue formation.^11^ Medical-grade TPUs have been used in implantable medical devices for decades, but recently, there has been an increasing interest in their application in tissue engineering, as they allow easier handling and suturing and they also possess good blood compatibility and resistance to microorganism colonization and infection.^12−14^

Moreover, inorganic materials have also gained attention in recent years to dope polymeric scaffolds. In fact, they have been widely used to enhance physiological and mechanical properties, with the consequence of forming scaffolds with the capability to guide and support the regeneration of different tissues.^15,16^ In particular, the use of metal oxides has been explored in recent years for various biomedical applications, as they exhibit unique physicochemical properties.^17^ Between the metal oxides, cerium oxide (CeO_2_) represents a potential tool to enhance the scaffold properties because numerous advantages of its use in tissue engineering have been recently highlighted, such as its role of protection toward different mammalian cell types (cartilage,^18^ neural,^19^ retinal,^20^ and cardiac^21^ cells) from oxidative stresses and inflammatory responses, in particular by scavenging reactive species, mitigating cytokine levels, and suppressing inflammation.^22,23^ Moreover, as mentioned, the mechanical properties are of fundamental importance for the orthopedic regeneration, but although numerous materials have been characterized to correspond with the tissues properties, numerous gaps are still present, leading to scaffold failure during the experimental animal studies and the preclinical trials.^24,25^ CeO_2_ represents a potential tool also to fill this gap because it has been demonstrated to be capable of enhancing the scaffolds’ mechanical properties, acting as a reinforcement due to its inclusion as a nanofiller into the polymeric matrix.^26,27^

On the basis of these premises, the aim of this work was the study and the development of tubular nanofibrous scaffolds based on TPU and CeO_2_ nanoparticles. The scaffolds were also enriched with a glycosaminoglycan, the chondroitin sulfate (CS), as it is a structural component of the ECM able to interact with positively charged bioactive molecules, in particular, growth factors, and effectively enhance the cell proliferation.^28,29^ In particular, the addition of CS is intended to stimulate the cell adhesion onto the scaffolds and consequently their proliferation, overcoming the TPU hydrophobicity.

The developed scaffold should emulate the structural and biomechanical functions of the ECM of the natural tendons, stimulating the host cell adhesion and proliferation and also guaranteeing a mechanical support against stresses during the entire regeneration process of the new tissue. Electrospinning has been used to obtain a nanofibrous structure. The nanofibrous scaffolds were characterized for physicochemical (morphology, solid-state and structural properties, mechanical behavior) and biopharmaceutical (in vitro degradation and in vitro and in vivo safety) properties.

## 2 Experimental Section

### 2.1 Materials

Polymers used were as follows: medical-grade thermoplastic polyurethane (TPU) (Pathway, Lubrizol Advanced Materials, New Milford, USA), chondroitin sulfate (CS), sodium bovine 100 EP (low molecular weight of 14 kDa), combination of chondroitin C (chondroitin 6 sulfate) and chondroitin A (chondroitin 4 sulfate), β-1,4-linked d-glucuronic acid, and β-1,3-linked *N*-acetyl galactosamine (Bioiberica, Barenz, Italy). The inorganic used was the cerium(IV) oxide nanopowder (particle size <25 nm) (Sigma-Aldrich, St. Louis, USA). The solvent used was acetic acid glacial (Carlo Erba Reagents, Val-de-Reuil Cedex, France).

### 2.2 Preparation of the Polymeric Blends

Table 1 shows the composition of the blends utilized to obtain the equivalent scaffolds. TPU was dissolved in water/acetic acid (1:9) at room temperature under magnetic stirring overnight. Initially, various percentages of TPU were tested to assess the polymer concentration influence on the electrospinning process and fiber morphology. The concentration that provided the formation of homogeneous nanofibers was chosen for the loading of CS and CeO_2_, alone and combined.

**Table 1:** Qualitative and Quantitative Composition of the Blends^a^

blend	TPU % (w/w)	CeO_2_ % (w/w)	CS % (w/w)
T6	6		
T9	9		
T12	12		
T12-CS	12		1
T12-CeO_2_	12	0.1	
T12-CS-CeO_2_	12	0.1	1

a All blends were prepared in water/acetic acid (1:9 volume ratio).

### 2.3 Development of the Electrospun Scaffolds

Scaffolds were developed by means of an electrospinning apparatus (STKIT-40, Linari Engineering, Pisa, Italy) equipped with a volumetric pump (Razel R99-E) and a high-voltage power supply (Razel R99-E 40 kV). Moreover, a 10 mL syringe with an inox needle (21G) was used. Two different types of collector, one static and flat, to obtain random scaffolds (R T12, R T12-CS, R T12-CeO_2_, and R T12-CS-CeO_2_) and a stainless steel cylindrical rotating drum (3 mm in diameter, 150 mm in length, and 0.3 mm in thickness) to obtain aligned tubular scaffolds (A T12, A T12-CS, A T12-CeO_2_, and A T12-CS-CeO_2_) were used. The distance between the collector and the tip of the needle was 20 cm, the flow rate was 0.758 mL/h, the voltage was 22 kV, and the rotation speed was 9600 rpm. The relative humidity was set at 20%, whereas the environmental temperature was 25 °C.

All the scaffolds were insoluble in water, and they were vacuum dried at 60 °C for 12 h to ensure the removal of any remaining solvent.^30^

### 2.4 Scaffolds’ Chemicophysical Characterization

The scaffolds’ morphology was assessed with a scanning electron microscope (SEM) (Tescan, Mira3XMU, Brno, Czech Republic) by sputtering the samples with graphite. The nanofibers’ dimensions were evaluated by an image analysis software (ImageJ, ICY, Institut Pasteur, Paris, France). For this purpose, to ensure that the measured fibers were randomly chosen and representative of the whole scaffold, three different images were used, and 30 analyses each were performed, with a final total of 90 analyses.

The incorporation of the CeO_2_ nanoparticles into the fibrous matrix was assessed with a transmission electron microscope (TEM) (JEOL JEM-1200 EX II microscope; CCD camera Olympus Mega View G2 with 1376 × 1032 pixel format, Tokyo, Japan; operating HV at 100 kV; magnification 100k). In this regard, the fibers were electrospun onto the grids (formavar/carbon 300 mesh Cu, Agar Scientific, Monterotondo (RM), Italy).

The wettability of the scaffolds was tested by contact angle measurements (DMe-211 Plus; FAMAS software, Kyowa, Osaka, Japan).

### 2.5 Structural Characterization

Fourier-transform infrared spectroscopy (FTIR) analysis was carried out using a JASCO 6200 apparatus (Tokyo, Japan) equipped with a Ge ATR. The spectra were recorded from 400 to 4000 cm^–1^ with a resolution of 2 cm^–1^, and the results were processed with a specific software (Spectra Manager v2). The noise was also removed with the Savitzky–Golay filter (OriginPro 2021b, OriginLab Corporation).

Thermogravimetric analysis (TGA) together with differential scanning calorimetry (DSC) was performed by means of a TGA/DSC1 equipment (Mettler-Toledo GMBH, Spain) equipped with a horizontal oven and a microbalance with 0.1 μg precision. The temperature range was from 25 to 950 °C, with the heating rate set at 10 °C/min in atmospheric air. About 20 mg of the sample was weighted in aluminum sample pans.

Small angle X-ray scattering spectra were recorded at the ID02 SAXS beamline of ESRF (Grenoble, France) (DOI: 10.15151/ESRF-ES-58593) using synchrotron light. Small pieces of scaffolds (0.5 × 0.5 cm) were cut, inserted in Kapton capillaries, and hydrated with water. The scattered intensity was measured in a wide range of momentum transfer *q*, 0.006 < *q* < 7.5 nm^–1^, where *q* = 4πsen(θ/2)/λ, where θ is the scattering angle and λ = 0.1 nm is the radiation wavelength. The intensity spectra were recorded putting the sample and the detector at two different distances (1 and 10 m) and bonded after meticulous background subtraction and angular regrouping.

### 2.6 Mechanical Property Evaluation

The scaffolds’ mechanical properties were evaluated by means of a dynamometer (TA-XT plus, Stable Microsystems, Italy) with a 5.0 kg load cell. The nanofibrous scaffolds were cut (pieces: 3 × 1 cm) and loaded between two tensile grips (A/TG probe; starting distance: 60.0 mm). The upper grip was moved constantly at 5.0 mm/s speed until the scaffold’s rupture. Mechanical properties were evaluated dry and after hydration, recording the force at break vs distance. Moreover, the percentage of elongation and the Young’s modulus were calculated.^31^

An analysis of the morphology during stimulation to mechanical stresses was also performed by SAXS. The samples were cut into rectangular strips of about 1 × 6 cm and mounted directly on the X-ray beamline, as shown in Figure S1 (Supporting Information), with a distance between the grips of 3.5 cm at rest. Measurements were performed at different elongations, up to 2.5 cm and at fixed elongation during dehydration. The scattered intensity was acquired on a 2D detector and visualized to observe asymmetries in the pattern.

### 2.7 Scaffolds’ In Vitro Degradation

To assess in vitro degradation, each scaffold (10 mg), randomly or aligned collected, was weighed and put at 37 °C in 4 mL of PBS (phosphate-buffered saline pH 7.4, Sigma-Aldrich, Milan, Italy). Samples were removed from the medium after 4, 8, and 12 weeks; washed twice in distilled water; dried in an oven at 60 °C for 1 h, ensuring their complete drying; and reweighed. The scaffolds’ weight loss (%) was calculated as the ratio between the weight after degradation and the initial weight.^32^ At every time interval (4, 8, and 12 weeks), the fibrous scaffolds’ structure was evaluated by means of SEM, as previously described (Section 2.4), and their mechanical properties were assessed using a dynamometer, as previously described (Section 2.6), to evaluate their changes during the degradation process.

#### 2.7.1 CS and CeO_2_ Release

The release of CS from the scaffolds was assayed using a CS ELISA kit (Aviva Systems Biology, San Diego, USA) for quantitative measurement of CS. Supernatants were collected from the in vitro degradation test after 4, 8, and 12 weeks, and the CS content was evaluated at 450 nm, setting 570 nm as wavelength correction. The method was linear with concentrations from 3 to 0.06 mg/mL, having an *R*^2^ of 0.9991. T12 and T12-CeO_2_ scaffolds were used as negative control.

For the evaluation of the CeO_2_ release from the scaffolds, the supernatants collected from the in vitro degradation test after 4, 8, and 12 weeks were filtered with a 0.22 μm filter; diluted in ultrapure water (1:5 volume ratio); and analyzed by inductively coupled plasma mass spectrometry (ICP-MS, Elan DRC-e, PerkinElmer, Shelton, CT, USA). T12 and T12-CS scaffolds were used as negative control.

### 2.8 Cell Proliferation Assay

The cell proliferation and viability were evaluated in vitro using normal human tenocytes (TEN-1) (fifth passage maximum; ZenBio, Durham, NC, USA). Tenocyte growth medium (ZenBio, Durham, USA) was prepared with 10% v/v of fetal bovine serum (FBS, Euroclone, Milan, Italy) and 200 IU/mL penicillin/0.2 mg/mL streptomycin (Sigma-Aldrich, Milan, Italy), and the flasks were coated with collagen (rat tail collagen coating solution, Cell Applications, Italy) before TEN-1 seeding. The cells were put into a CO_2_ incubator (PBI International, Milano, Italy) with a temperature of 37 °C and 5% CO_2_ atmosphere (relative humidity (RH): 95%).

Before testing, the scaffolds (5 mm diameter, 0.2 mm thickness) were placed in a 96-well plate and sterilized by UV radiation for 20 min. Afterward, TEN-1 cells were seeded onto the scaffolds with 2 × 10^4^ cells/well density and reincubated. The positive control was represented by TEN-1 grown in standard conditions (growth medium, GM). Moreover, CeO_2_ colloidal suspension (using the same metal oxide amount as the nanofibrous scaffolds) was also tested. After 7, 14, and 21 days of contact with the scaffolds, 100 μL of Alamar Blue (10% (v/v); AlamarBlue HS cell viability reagent, Invitrogen, Thermo Fisher, Monza, Italy) was added into the wells. The Alamar Blue was incubated for 3 h at 37 °C in the dark and then withdrawn and transferred in a new plate. Each well was refilled with fresh medium to continue the culture. The fluorescence intensity (FI) of the Alamar Blue was measured (FLUOstar Omega, BMG LABTECH, Aylesbury, UK) at λex = 530 nm and λem = 590 nm. FI is directly related to cell viability.

### 2.9 Cell Morphology

The cell morphology after 21 days of contact with the scaffolds was assessed by means of confocal laser scanning microscopy (CLSM). Cells were fixed using a glutaraldehyde solution (3% (v/v)) for 2 h. Afterward, the substrates were washed three times with PBS. The cell nucleus was stained with propidium iodide (red, Sigma-Aldrich, Milano, Italy; 50 μL/sample at 25 μg/mL in PBS in each well, contact time 2 min), whereas the cytoskeleton was stained using FITC Atto 488 phalloidin (green, Sigma-Aldrich, Milan, Italy; 50 μL at 20 μg/mL in PBS in each well, contact time 40 min). The scaffold images were acquired with a Leica CLSM (TCS SP2, Leica Microsystems, Buccinasco (MI), Italy) with the following settings: λex = 535 nm and λem = 617 nm (propidium iodide) and λex = 501 nm and λem = 523 nm (FITC-phalloidin). The images were processed with the Leica Microsystem software (Buccinasco (MI), Italy).

### 2.10 In Vivo Evaluation of System Safety

The animal experiments were compliant with the European Communities Council Directive 2010/63/EU. The protocol was approved by the University of Pavia and ISS. Nine male Wistar rats (weight: 200–250 g, Envigo RMS S.r.l.) were anesthetized with equitensine at 3 mL/kg, and their backs were shaved to remove all hair, as described by Ruggeri et al.^33^ After back shaving, 5 mm diameter scaffolds were subcutaneously implanted. Eighteen days after the treatment, full thickness biopsies were taken, and histological analysis was performed. Biopsies of intact skin and of the wound treated with a saline solution were also taken for comparison as controls.

#### 2.10.1 Histological Analysis

As described by Ruggeri et al.,^33^ tissue samples were fixed (4% neutral buffered formalin) and embedded in paraffin. Five micrometer sections were prepared, and hematoxylin and eosin (H&E) and picrosirius red (PSR) staining was performed. For PSR staining, deparaffinized sections were hydrated, stained with Weigert’s hematoxylin (nuclei), and then stained with PSR for 60 min. Afterward, the sections were observed using a light microscope (Carl Zeiss Axiophot) and imaged (Nikon DS-Fi2).

### 2.11 Statistical Analysis

Statistical evaluation was performed using one-way ANOVA followed by post hoc Scheffé test (Astatsa statistical calculator). *p* < 0.05 was considered significant.

## 3 Results and Discussion

### 3.1 Scaffolds’ Chemicophysical Characterization

**Figure 1:**
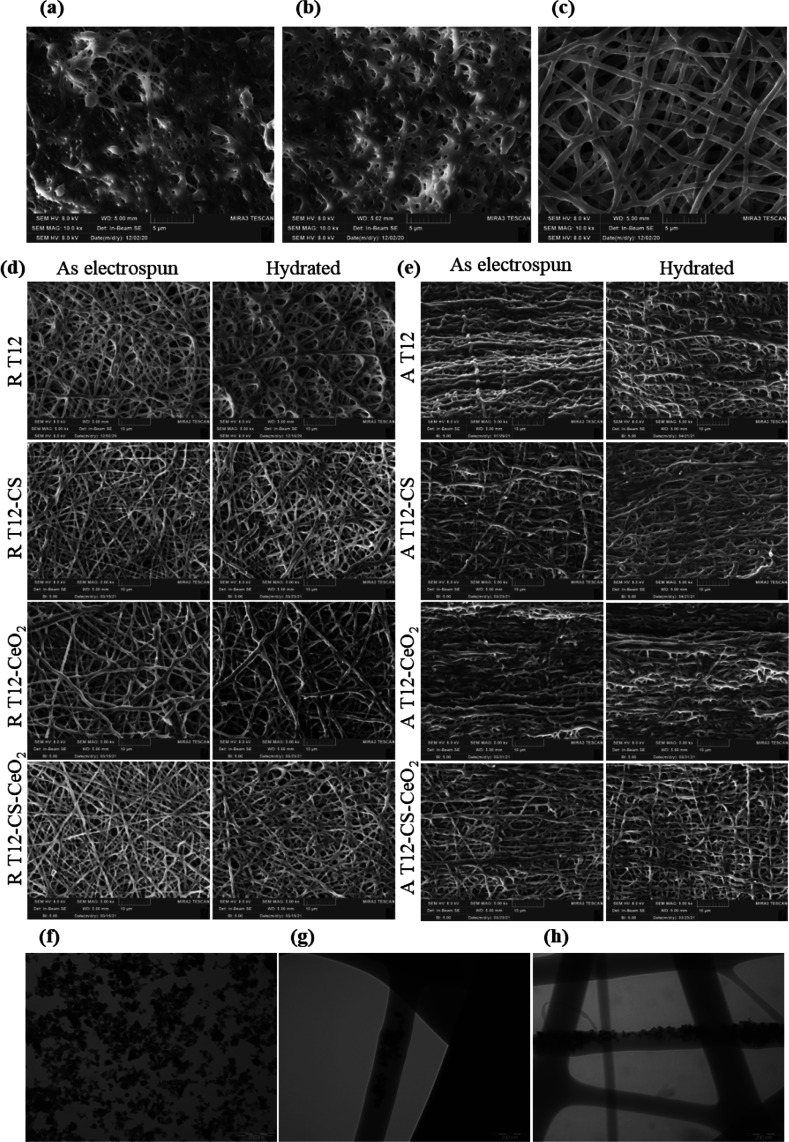
SEM micrographs of (a) T6 (dimensional analysis: 351.57 ± 98.02 nm), (b) T9 (dimensional analysis: 349.80 ± 78.72 nm), and (c) T12 (dimensional analysis: 391.17 ± 50.34 nm) electrospun fibers at 10.0k× magnification. (d) SEM micrographs of random T12, T12-CS, T12-CeO_2_, and T12-CS-CeO_2_ as electrospun (dimensional analysis: 430.17 ± 50.34, 402.07 ± 76.31, 566.60 ± 67.35, and 407.17 ± 36.62 nm, respectively) and after 6 days of hydration (dimensional analysis: 439.93 ± 47.13, 435.70 ± 92.08, 595.43 ± 92.08, and 439.77 ± 44.79 nm, respectively; % of fiber increase: 2.27 ± 0.50, 8.36 ± 1.10, 5.09 ± 0.90, and 8.01 ± 1.03%, respectively) at 5.0k× magnification. (e) SEM micrographs of aligned tubular T12, T12-CS, T12-CeO_2_, and T12-CS-CeO_2_ as electrospun (dimensional analysis: 505.81 ± 93.27, 433.27 ± 95.80, 518.07 ± 91.56, and 439.53 ± 67.92 nm) and after 6 days of hydration (dimensional analysis: 521.30 ± 96.11, 476.27 ± 114.67, 536.53 ± 107.66, and 468.80 ± 93.83 nm, respectively; % of fiber increase: 3.06 ± 0.70, 9.92 ± 1.40, 3.56 ± 0.60, and 6.66 ± 1.10%, respectively) at 5.0k× magnification. TEM images of (f) CeO_2_ powder, (g) T12-CeO_2_ scaffold, and (h) T12-CS-CeO_2_ scaffold at 100k× magnification. Scale bar: 200 nm (mean values ± s.d.; *n* = 90).

A preformulative study was performed to investigate the suitable concentration to obtain a continuous polymeric jet. Six percent w/w TPU was the threshold; however, 12% w/w was the minimum concentration to obtain the formation of regular and homogenous fine nanofibers without defects such as beads and knots. Figure 1a–c reports the SEM micrographs of the preformulative study fibers (T6, T9, and T12). The TPU concentration affects the fibers’ morphology: lower concentrations (6 and 9% w/w) produce merged and non-homogeneous fibers, whereas the higher concentration (12% w/w) produces regular fibers without beads. This could be related to the polymeric blends’ surface tension, conductivity, and consistency that indeed play a pivotal role in the electrospinning process. All the TPU polymeric blends are characterized by similar values of surface tension, whereas their consistency and conductivity are directly related to the polymer concentration (see Supporting Information (SI) Table S1). In fact, the surface tension affects the formation of Taylor’s cone, whereas the blend conductivity and consistency favor the electrospinning process and, consequently, the formation of homogeneous fibers without defects.^33,34^

As the T12 blend allowed obtaining nanofibers with better morphological characteristics, it was selected for the loading of CS and CeO_2_ nanoparticles, alone and combined, to increase the scaffolds’ mechanical properties and promote the cell adhesion and growth.

The morphology of the scaffolds was investigated upon hydration of randomly or aligned structure. Figure 1d reports SEM micrographs of the scaffolds electrospun in random conformation (with a flat collector) and doped with CS or CeO_2_ or the combination of the two. The analysis was performed in the moment after the electrospinning process and also after 6 days of hydration in PBS (Sigma, Milan, Italy). The doping with the active components does not affect the electrospinning process, and the morphological analysis evidences that all the scaffolds are characterized by a nanofibrous structure even after 6 days of hydration. Moreover, the T12 fibers show a smooth surface, whereas the scaffolds doped with CS and CeO_2_ are characterized by a moderate rough surface, which has been demonstrated to promote the initial cell anchorage to the fibers, followed by spreading and proliferation.^35^ Further, the EDX study confirms the presence of CeO_2_ and CS in the TPU matrix as the T12-CS spectrum shows the presence of sulfur (wt % 4.39), the T12-CeO_2_ spectrum shows the presence of cerium (wt % 0.81), whereas the T12-CS-CeO_2_ spectrum shows the presence of both sulfur and cerium (wt % 0.23 and 0.61, respectively) compared to the carbon and oxygen alone of the T12 spectrum (SI Figure S2).

The doping does not significantly change the nanofiber dimensions that are conceivably determined by the polymer properties (SI Table S1) rather than the added components.

Figure 1e reports SEM micrographs of the scaffolds electrospun using the cylindrical rotating drum to obtain aligned fibers with a structure similar to the tendon hierarchical one. The fibers have nanometric dimensions and an aligned orientation. Even in this case, the T12 fibers show a smooth surface, whereas the scaffolds loaded with CS and CeO_2_ are characterized by a rough surface. After 6 days of hydration, the morphology does not change, and only a slight swelling is distinguishable.

The identification of CeO_2_ nanoparticles into the fibrous matrix was studied using TEM. Figure 1f–h reports the TEM images of T12-CeO_2_ and T12-CS-CeO_2_ electrospun fibers compared to the CeO_2_ powder alone. It is clearly noticeable that CeO_2_ nanoparticles are incorporated into the singular fibers, affecting the surface roughness and shape. Conversely, the CS presence is not visible into the fibrous structure, suggesting that the CS chains are homogeneously interlaced with the TPU ones.

**Figure 2:**
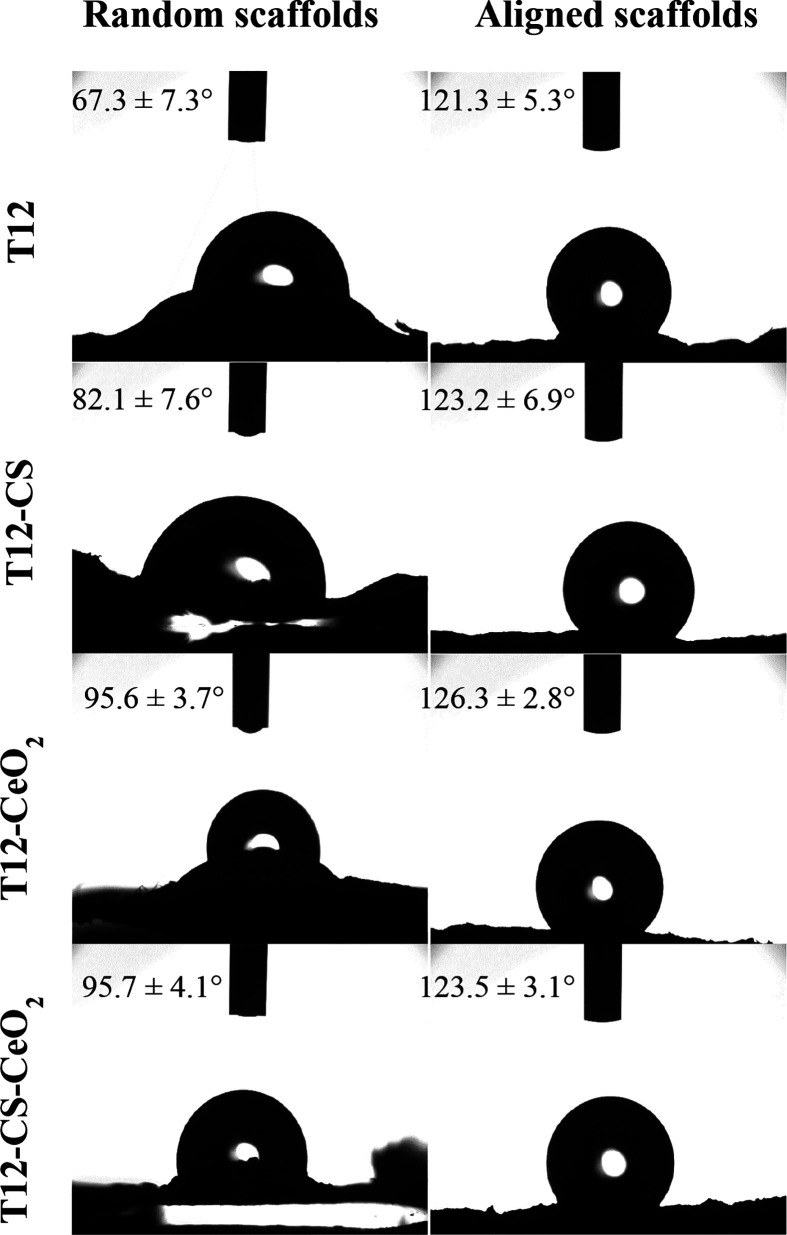
Images of the buffer after 1000 ms of contact with the scaffolds’ surface. In each image, the contact angle value is reported (mean values ± s.d.; *n* = 3) (needle diameter = 0.405 mm).

The interfacial properties of the scaffolds and their hydrophilicity and wettability were evaluated using contact angle measurements. Figure 2 reports the shape and the contact angle values for a 0.4 μL buffer drop released onto the scaffolds in both the aligned tubular and random structures.

It is evident that the fiber alignment regulates the surface wettability despite the same polymeric composition. In particular, the random scaffolds present higher wettability, which decreases in the presence of CeO_2_ because of its intrinsic hydrophobicity.^36^ The contact angle values of the aligned scaffolds, which are higher than those of the random ones, could be imputable to the fiber organization that influences the water spreading onto the scaffold surface. In particular, aligned nanofibers are characterized by interfiber spacing able to generate capillary-like forces parallel to fiber orientation, consequently preventing the spread of water in the opposite direction. On the other hand, when the scaffolds are in random shape, the boundary-induced forces induced by adjacent nanofibers are randomly directed without any influence on hydration.^37,38^

### 3.2 Structural Characterization

**Figure 3:**
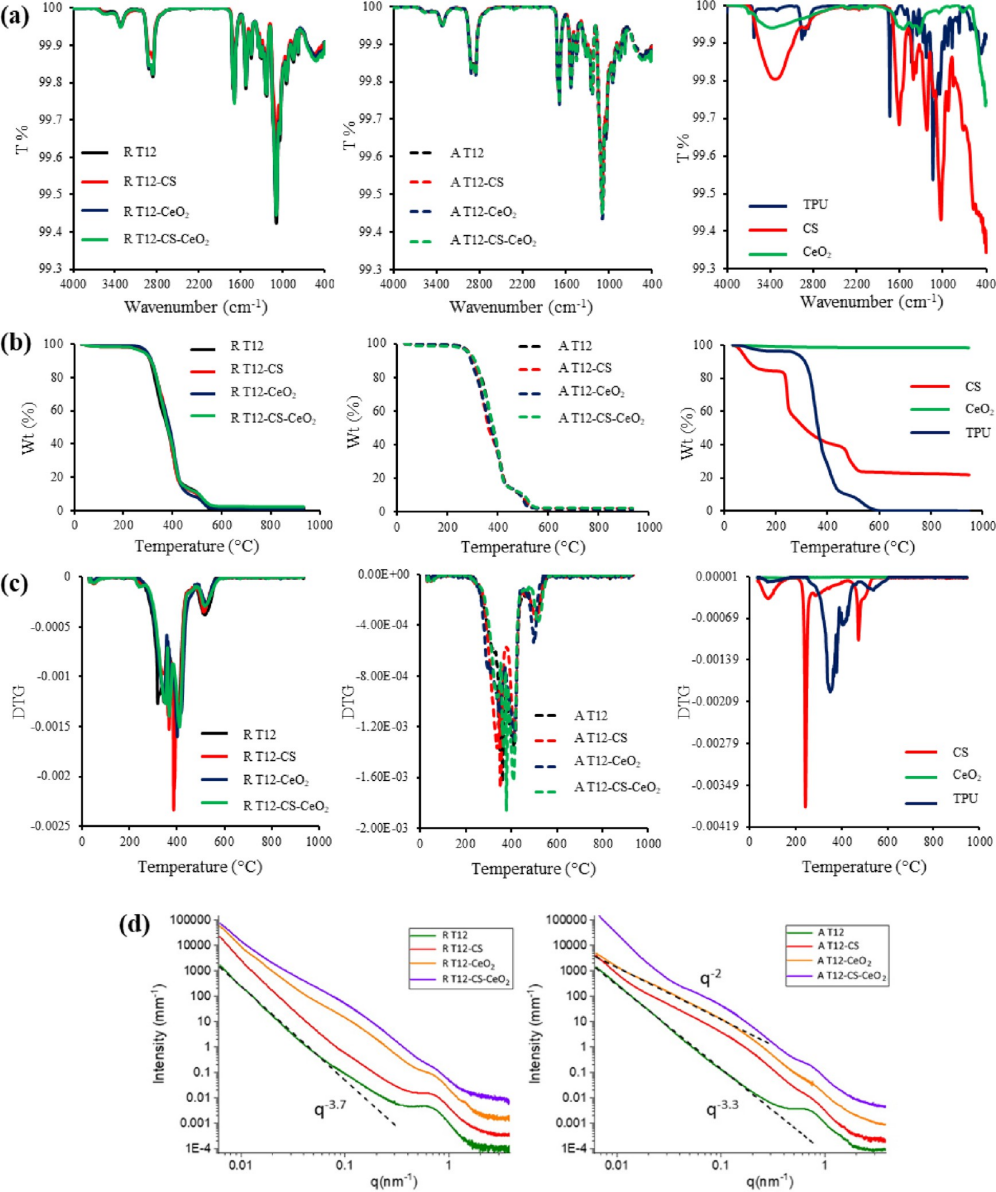
(a) FTIR spectra of the electrospun scaffolds random (R) and aligned (A) and of the raw materials (right column). (b) TGA analysis of the R and A scaffolds and of the raw materials (right column). (c) DSC analysis of the R and A scaffolds and of the raw materials (right column). (d) SAXS spectra of the electrospun scaffolds random (R) and aligned (A) vertically shifted for better visibility. Dashed lines represent the trend of the *I*(*q*) decay.

The scaffolds were studied by means of infrared spectroscopy, thermal analysis, and structural characterizations. Figure 3a reports the FTIR profiles of the electrospun scaffolds random (R) and aligned (A), Figure 3b reports the TGA analysis, and Figure 3c reports the DSC analysis. The FTIR spectra arecharacteristic of the TPU, as it is the main component of the systems (Figure 3a, right panel). Moreover, all scaffolds independently of their composition and of the organization of the fibers are stable until 250 °C (Figure 3b), whereas between 250 and 300 °C, they show an intense weight loss. The same trend is observed in the DSC profiles (Figure 3c), where at temperatures higher than 250 °C, other endothermic peaks are present conceivably related to the component degradation.

The architecture of scaffolds and fibers on the length scale between 100 and 1 nm was investigated by small angle X-ray scattering (SAXS). SAXS spectra of electrospun scaffolds (random R and aligned A) are reported in Figure 3d.

All the reported scattering profiles show a low-*q* scattering feature dominated by a power-law slope, I(*q*) ÷ *q*^–s^, and a high-*q* broad peak at *q* ≅ 0.7 nm^–1^, corresponding to a characteristic distance *d* = 2π/*q* = 9 nm. This peak is characteristic of the phase-separated hard and soft domains formed by the arrangement on the nanoscale of TPU block copolymers composed of alternating flexible soft segments and rigid, hard segments.^39,40^ The typical local arrangement of TPUs is preserved after random and aligned electrospinning processes and in the presence of CS, CeO_2_, and CS-CeO_2_, although it is less defined in the presence of CeO_2_ nanoparticles.

In the low-*q* region, *q* < 0.04 nm^–1^, corresponding to lengths longer than 150 nm, the intensity decays of the different scaffolds follow an *I*(*q*) ÷ *q*^–s^ power law. For random R scaffolds, the exponent, dependent on the fractal dimension, is 3.7 < *s* < 4, revealing a surface fractal arrangement of the fibers with intermediate to negligible roughness.

Doping with CS does not affect the internal arrangement of R scaffolds, suggesting that CS chains could have been homogeneously distributed in the TPU fibrous matrix, adopting the same arrangement on the whole scale of lengths.

The presence of CeO_2_ nanoparticles affects the intensity profiles of R scaffolds in the 0.03–0.3 nm^–1^*q* region, as visible in Figure 3d, confirming that CeO_2_ nanoparticles are hosted and dispersed into the fibrous matrix.

The internal structure of aligned A scaffolds is quite similar to the one of R scaffolds in the case of T12, although it shows a less steep intensity decay slope, indicating an increase in the roughness of the surface. Interestingly, the doped fibrous matrix adopts an arrangement characterized by an *I*(*q*) ÷ *q*^–2^ power law in the region 0.03–0.15 nm^–1^. The combined rotation and horizontal oscillation of the cylindrical rotating collector during the electrospinning process, with a dragging effect also on the needle, induce an alignment of the fibers while affecting their roughness and internal structure on the length scale of the tens of nanometers. Results suggest a higher roughness of the fiber surface and a looser packing of polymer chains within the fiber.

### 3.3 Mechanical Property Evaluation

**Figure 4:**
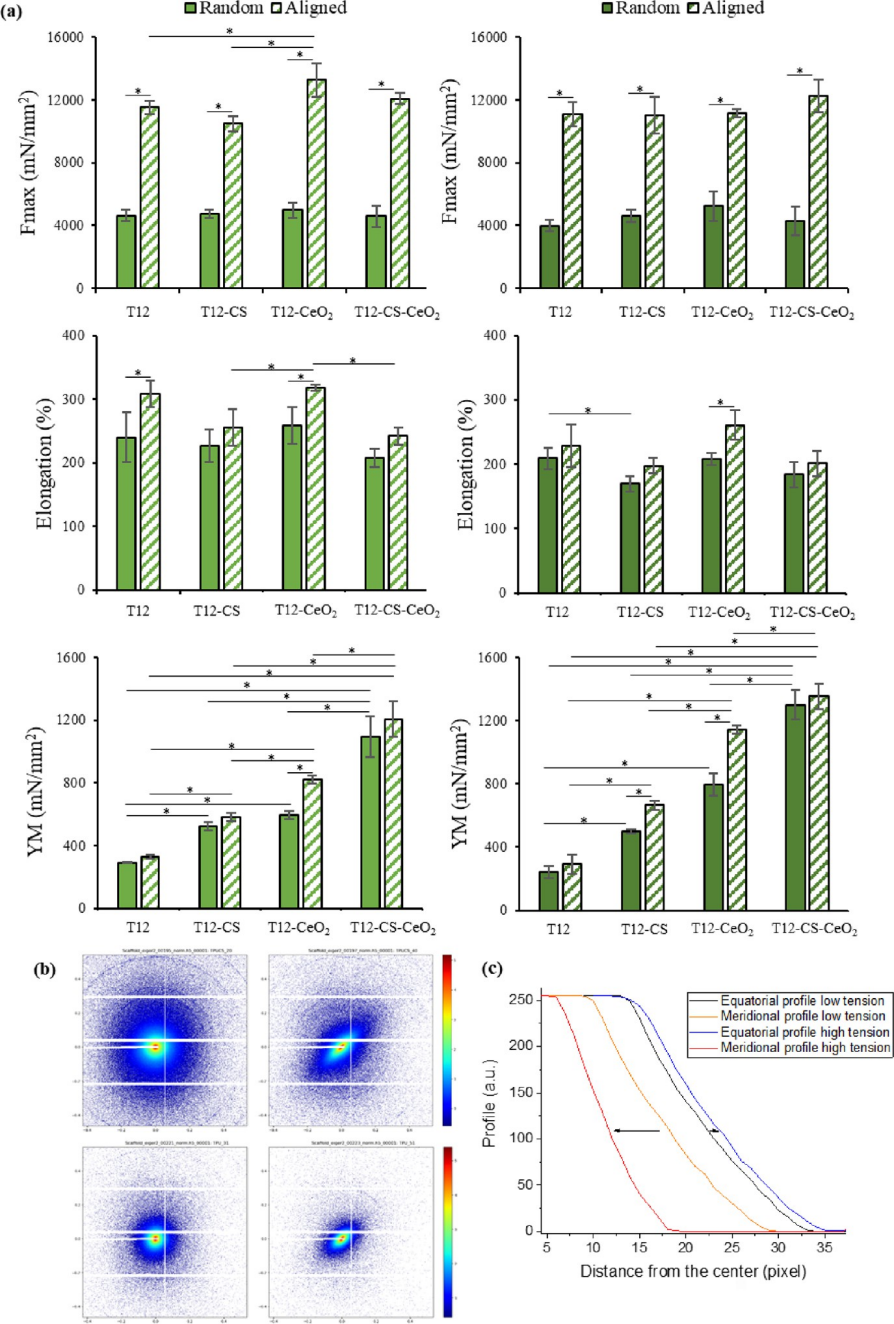
(a) Mechanical properties of random (full color) and tubular aligned (shaded) scaffolds in both dry (left column) and hydrated (right column) states (mean values ± s.d.; *n* = 6). * indicates statistical differences between results. (b) SAXS patterns of random R scaffolds (T12-CS upper line, T12 bottom line) at equilibrium (left) and at 170% elongation (right) in dry conditions. (c) Equatorial and meridional intensity profiles as extracted from T12 2D SAXS patterns measured at different elongations.

It is known that the scaffolds’ mechanical properties could be important to affect cell differentiation and regenerative functions because the mechanical properties of the ECM environment have the capability to influence intracellular signaling and cell response.^41^ The mechanical properties of the scaffolds were assessed by means of tensile measurement, and the structural evolution on the mesoscale upon tension was investigated using SAXS. Figure 4 reports the mechanical properties of random and tubular aligned scaffolds in both dry (left column) and hydrated (right column) states. As a general trend, the results demonstrate that the tubular aligned scaffolds are characterized by remarkable mechanical properties, higher than their random counterparts. This could be reconducted to the fibers’ alignment in the tensile load direction, which could represent an effective method to mimic the tendon fascicles and to improve the scaffolds’ capability to withstand the mechanical loads and to decrease the structure deformation.^42^ In particular, the tubular scaffolds are able to withstand higher stress and larger deformation before breaking. Moreover, the presence of CeO_2_ notably increased the Fmax of the scaffolds as a result of the CeO_2_ intrinsic hardness.^43^ Importantly, the scaffolds loaded with CeO_2_ reach mechanical properties, both elongation and force at break, similar to those of the native tendons. In fact, the mechanical properties of different types of human tendons and ligaments vary according to their location, with an ultimate tensile strength that ranges from 5 to 100 MPa and a strain of failure between 10 and 15%.^44^ For this reason, the tubular T12-CeO_2_ and T12-CS-CeO_2_ scaffolds, which possess an ultimate tensile stress of 13.27 and 12.09 MPa, respectively, and a strain failure of about 300 and 250%, respectively, represent excellent candidates to substitute tendon tissue because they guarantee to sustain a 15% elongation (the strain of failure of native tendons) without breaking. Finally, the tendon stiffness greatly influences the tendon YM in vivo, depending on the musculoskeletal functions. Similarly, the YM of the scaffold object of this study could be modulated by increasing or reducing their stiffness and even combining different scaffolds to adapt the corresponding YM to the one required. The increase in the scaffolds’ Fmax could be attributed to an increase in rigidity with respect to the pure TPU when the CeO_2_ is added due to the resulting adhesion between the two materials. The CeO_2_ nanoparticles’ homogeneous dispersion could facilitate a uniform distribution of the mechanical stress, resulting in an improvement of the tensile strength. In fact, CeO_2_ in adequate concentrations could act as a nanofiller, filling the voids in the singular polymeric fibers and resulting in higher degrees of stress transfer and, therefore, higher tensile strength and modulus.^45,46^

Moreover, TPUs are considered as resilient elastomers, which possess a range of desirable properties such as elastomeric property and resistance to abrasion.^47,48^ Because of their elastomeric properties, TPU is an excellent material to build shape-memory scaffolds, with the ability to recover the original shape after being subjected to mechanical stresses until they break, as seen by Ahmad et al.^49^ This trend was also observed in the scaffolds developed in this work. In fact, the scaffolds are able to return to their original shape after mechanical tests when breaking did not occur. This is due to the hard segments of the TPU chains because they contain long sequences of hydrogen bonding sites that serve as the physical cross-links. These cross-link points prevent close chains slipping from each other when subjected to deformation, acting as a fixed phase during the shape recovery process.^50^

As a proof of concept, structural characterization by SAXS on the scaffolds submitted to mechanical deformation was performed. The 2D patterns for T12 and T12-CS dry R scaffold at 0 and 170% elongation are reported in Figure 4b. The patterns have been acquired on the SAXS detector at 10 m sample-to-detector distance, in the *q* < 0.5 nm^–1^ region, corresponding to a length scale of the order of hundreds of nanometers. Upon elongation, the scattered intensity patterns become asymmetric, along an axis tilted at 45°, the same value as the angular tilt of the tensiometer. The intensity profiles obtained on the equatorial and meridional directions are reported in Figure 4c. The different decays observed in elongated samples indicate an asymmetry in the internal structure of stretched fibers on the hundreds of nanometers length scale. The meridional profile shifts dramatically to low pixels, corresponding to low *q* values and longer lengths, with a shrinkage of *q* of about 170–180%, indicating that variation of the fiber structure on the mesoscale corresponds to the macroscopic elongation imposed to the scaffold. The equatorial profile, perpendicular to the stretch direction, slightly shifts to high pixels, corresponding to high *q* values and short lengths. For an elastic material subjected to stretching, the elongation along the tensile direction corresponds to a contraction in the other two dimensions (perpendicular to the tensile direction), which varies according to whether the volume of the material is conserved or not. The observed *q* expansion of the equatorial profile corresponds to a mean contraction along the other dimensions of the scaffold lower (115%) than expected for a constant volume (130%). This result indicates that the scaffold structure rearranges on the mesoscale when stretched. The process is reversible, and the scattering pattern recovers the initial symmetry when the stress is removed.

The structural properties of elongated R scaffolds changed in wet conditions, as can be observed in Figure S3 (SI), where the intensity 2D patterns have been measured during sample dehydration at fixed elongation (about 200%). The scattered intensity increase observed during dehydration is due to the higher contrast of fibers with respect to the air instead of water. The patterns become more asymmetric upon dehydration, revealing a different rearrangement of the structure on the hundreds of nanometers length scale when submitted to a fixed elongation as a function of water content.

### 3.4 Scaffolds’ In Vitro Degradation

**Figure 5:**
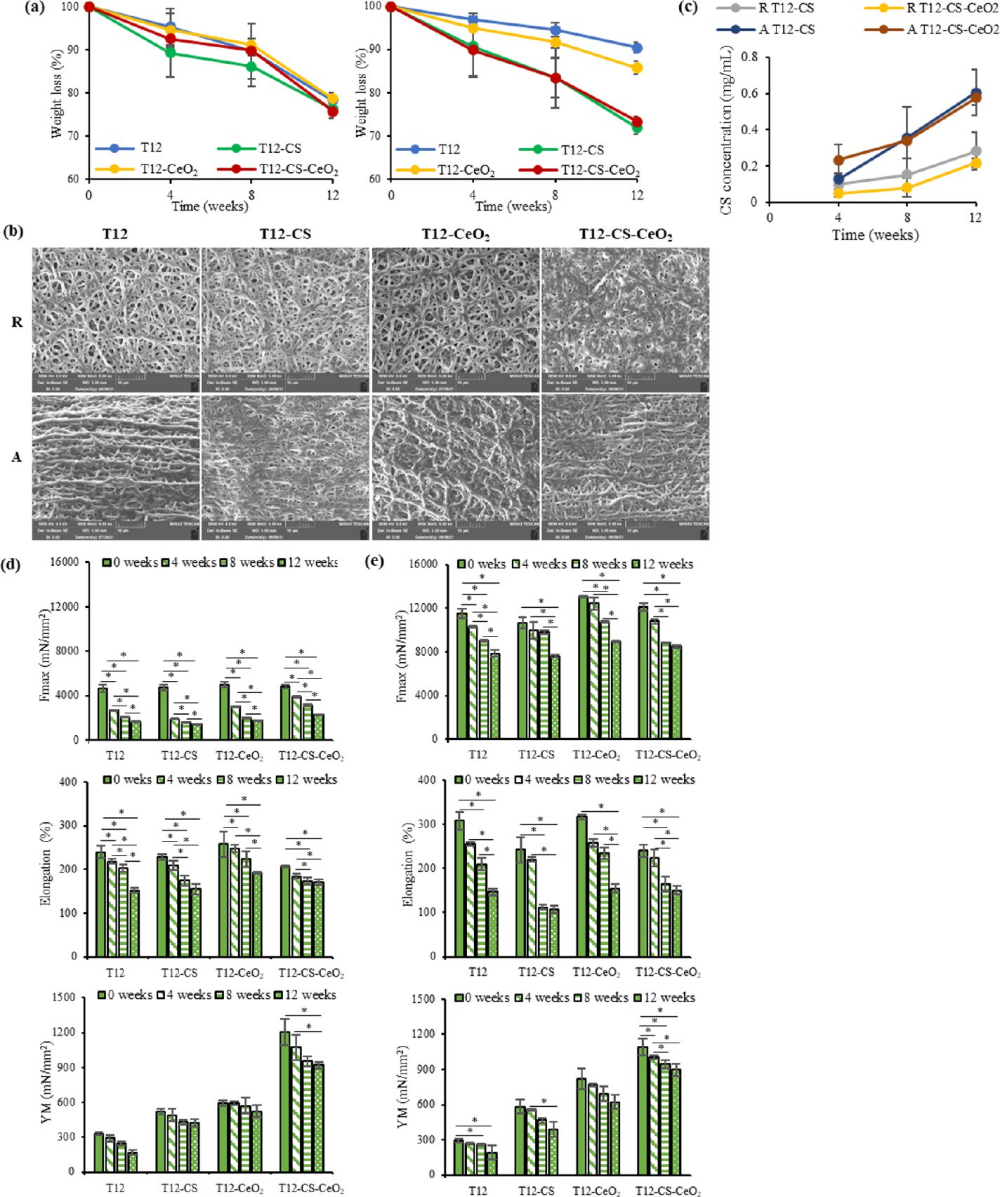
(a) Weight loss (%) evaluated for T12, T12-CS, T12-CeO_2_, and T12-CS-CeO_2_ scaffolds in both random (left panel) and tubular aligned (right panel) configuration (mean values ± s.d.; *n* = 4). (b) SEM micrographs of T12, T12-CS, T12-CeO_2_, and T12-CS-CeO_2_ scaffolds, random (R; dimensional analysis: 508.81 ± 98.05, 601.80 ± 133.52, 691.90 ± 134.09, and 713.17 ± 138.80 nm, respectively; % of fiber increase: 18.28 ± 1.20, 49.67 ± 2.30, 22.11 ± 1.70, and 75.15 ± 4.50%, respectively) and aligned (A; dimensional analysis: 873.80 ± 167.57, 670.43 ± 115.33, 766.03 ± 136.70, and 774.73 ± 134.10 nm, respectively; % of fiber increase: 72.75 ± 8.30, 54.74 ± 6.10, 47.86 ± 9.21, and 76.26 ± 9.40%, respectively), after 12 weeks of degradation at 5.0k× magnification (mean values ± s.d.; *n* = 90). (c) CS release from T12-CS and T12-CS-CeO_2_ scaffolds in both random (R) and tubular aligned (A) configuration (mean values ± s.d.; *n* = 4). Mechanical properties (Fmax, elongation, and YM) of (d) random and (e) tubular aligned scaffolds after 0, 4, 8, and 12 weeks of degradation (mean values ± s.d.; *n* = 6). * indicates statistical differences between results.

The degradation was investigated in vitro in PSB to simulate the pH and ionic strength of the aqueous environment, the site of scaffold implant. Figure 5a reports the scaffolds’, random (left panel) and tubular aligned (right panel), in vitro degradation in PBS at 37 °C. All the scaffolds are characterized by almost the same behavior independently of the composition. The scaffolds show a weight loss of about 5 (the aligned tubular) and 8% (the random) after 4 weeks, 10 (the aligned tubular) and 12% (the random) after 8 weeks, and between 15 and 29% (the aligned tubular) and 23% (the random) after 12 weeks in PBS. The higher degradability of the random scaffolds seems attributable to their higher wettability and higher porosity, as suggested by Luckachan and Pillai.^51^ However, the scaffolds maintain their integrity (fibrous morphology), although a slight increase in fiber diameter is recorded, as shown in the SEM micrographs where the % increase (%i) of dimensions is reported in the figure’s caption (Figure 5b). In particular, T12 and T12-CeO_2_ scaffolds are characterized by the lowest weight loss, in accordance with the lower wettability of their surface, whereas the scaffolds loaded with CS are characterized by a higher degradation degree after 12 weeks. The high hydrophilicity of CS seems to have a role in fiber swelling and in their degradation. Moreover, the evaluation of the CS release (Figure 5c) shows that the CS is slowly released from the scaffolds during the degradation. In particular, R T12-CS and R T12-CS-CeO_2_ scaffolds are characterized by a CS release of about 6% after 4 weeks, 8% after 8 weeks, and 20% after 12 weeks, whereas A T12-CS and A T12-CS-CeO_2_ scaffolds are characterized by a CS release of about 15% after 4 weeks, 30% after 8 weeks, and 50% after 12 weeks. This could be related to the higher presence of CS on the surface of the aligned scaffolds, which could also lead to a higher cell adhesion and proliferation. Moreover, it could be also related to the higher weight loss of A T12-CS and A T12-CS-CeO_2_ scaffolds with respect to A T12 and A T12-CeO_2_. On the other hand, no significant release of CeO_2_ is observed in the scaffolds during the degradation, as shown in Figure 5d. In fact, no significant differences are observed between the scaffolds and the time intervals, as they are all characterized by a CeO_2_ detection of <1 ppb. This could be related to the hydrophobic nature of CeO_2_, which remains strictly entrapped into the polymer matrix that was filtered before the cerium analysis and consequently could not be detected into the solution. This could mean that the component is well integrated into the polymeric matrix, acting as a mechanical property enhancer during the entire regeneration process, as TEM analysis suggests.

As a matter of fact, Figures 5d,e reports the mechanical properties tested at 0, 4, 8, and 12 weeks of degradation. All scaffolds show a linear decrease in time of the mechanical properties. The decrease is lower for the aligned tubular scaffolds, which maintain remarkable values of Fmax even after 12 weeks of degradation. The results could suggest a slow degradation in time of the scaffolds that should guarantee adequate mechanical support during the prolonged regeneration time of the tendon tissue. In particular, the tendon healing starts with an early inflammatory phase of about 1 week followed by a proliferative phase and by a remodeling phase, which last about 4 weeks and many months, respectively.^52,53^ For this reason, scaffolds with the capability of maintaining adequate morphology and mechanical properties for prolonged times are of fundamental importance.

### 3.5 Cell Proliferation Assay

**Figure 6:**
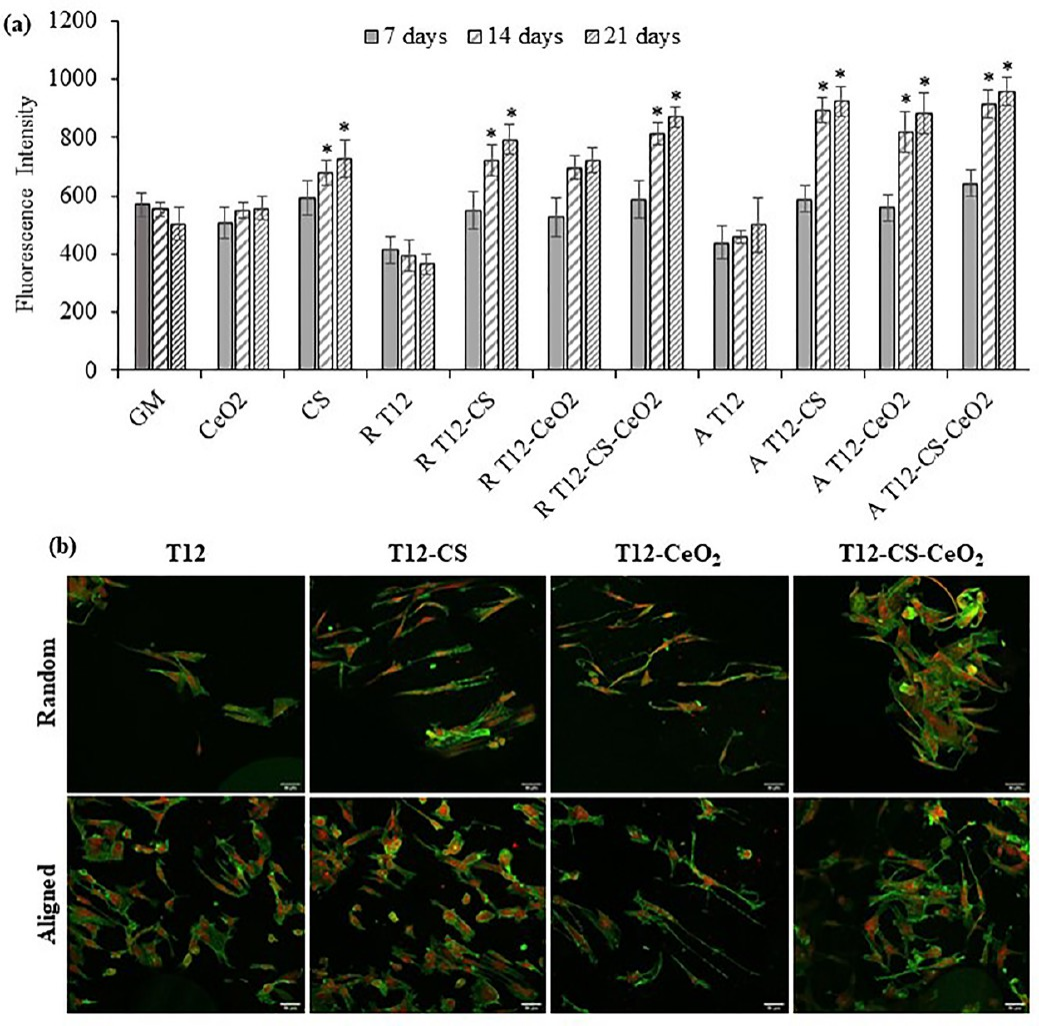
(a) Alamar blue assay evaluated for T12, T12-CS, T12-CeO_2_, and T12-CS-CeO_2_ scaffolds in both random (R) and tubular aligned (A) configurations (mean values ± s.d.; *n* = 6). (b) CLSM images of TEN-1 grown onto the random (R, upper row) and aligned (A, lower row) scaffolds after 21 days. Cytoskeletons stained in green; nuclei stained in red (scale bar: 50 μm). * indicates statistical differences with the positive control GM.

The cytocompatibility was assessed in vitro using normal human tenocytes (in vitro safety). Figure 6a reports the results in florescence intensity, FI of the Alamar blue assay, performed on both random and tubular aligned scaffolds after 7, 14, and 21 days of TEN-1 culture. The proliferation was compared to the cells’ growth in standard conditions (GM) and to the cells’ growth in contact with CeO_2_ and CS powders alone in the same quantity contained in the scaffolds (as control). The CeO_2_ nanoparticles prove to be safe and nontoxic for the cells, as they are capable to grow similarly to the GM. Moreover, CS proves to enhance the cell proliferation as it is characterized by a cell growth higher than that of the control. The scaffolds doped with the active components are also characterized by a cell growth higher than that of the control (cell growth in standard conditions). The tubular aligned scaffolds seem to perform better. Notably, the scaffolds loaded with CS show a significantly higher cell growth. The CS seems also to have a synergic effect with the CeO_2_, as the aligned scaffolds doped with both the active components are characterized by the best performance on cell proliferation. In fact, CS is well known as a facilitator of cell adhesion and an enhancer of their proliferation.^54^ In particular, CS is reported as able to increase the proliferation of fibroblasts and also collagen density, as it is a type of glycosaminoglican (GAG) that is found abundantly among other GAG in the body.^55^ On the other hand, CeO_2_ proved to improve the cell adhesion and proliferation as a result of its antioxidative properties that act as oxygen buffer.^56^ Notably, these could provide a better microenvironment for cells than undoped scaffolds.^57^

### 3.6 Cell Morphology

The cell morphology after 21 days of culture of tenocytes onto the scaffolds was assessed using CLSM. Figure 6b reports the CLSM images of the TEN-1 cells adhered onto the scaffold surface. TEN-1 cells grow more preferably onto the aligned scaffolds than the random ones. Moreover, the presence of CS enhances the cell adhesion and proliferation in both the random and aligned scaffolds, representing an interesting tool to effectively promote cell proliferation.

### 3.7 In Vivo Evaluation of System Safety

**Figure 7:**
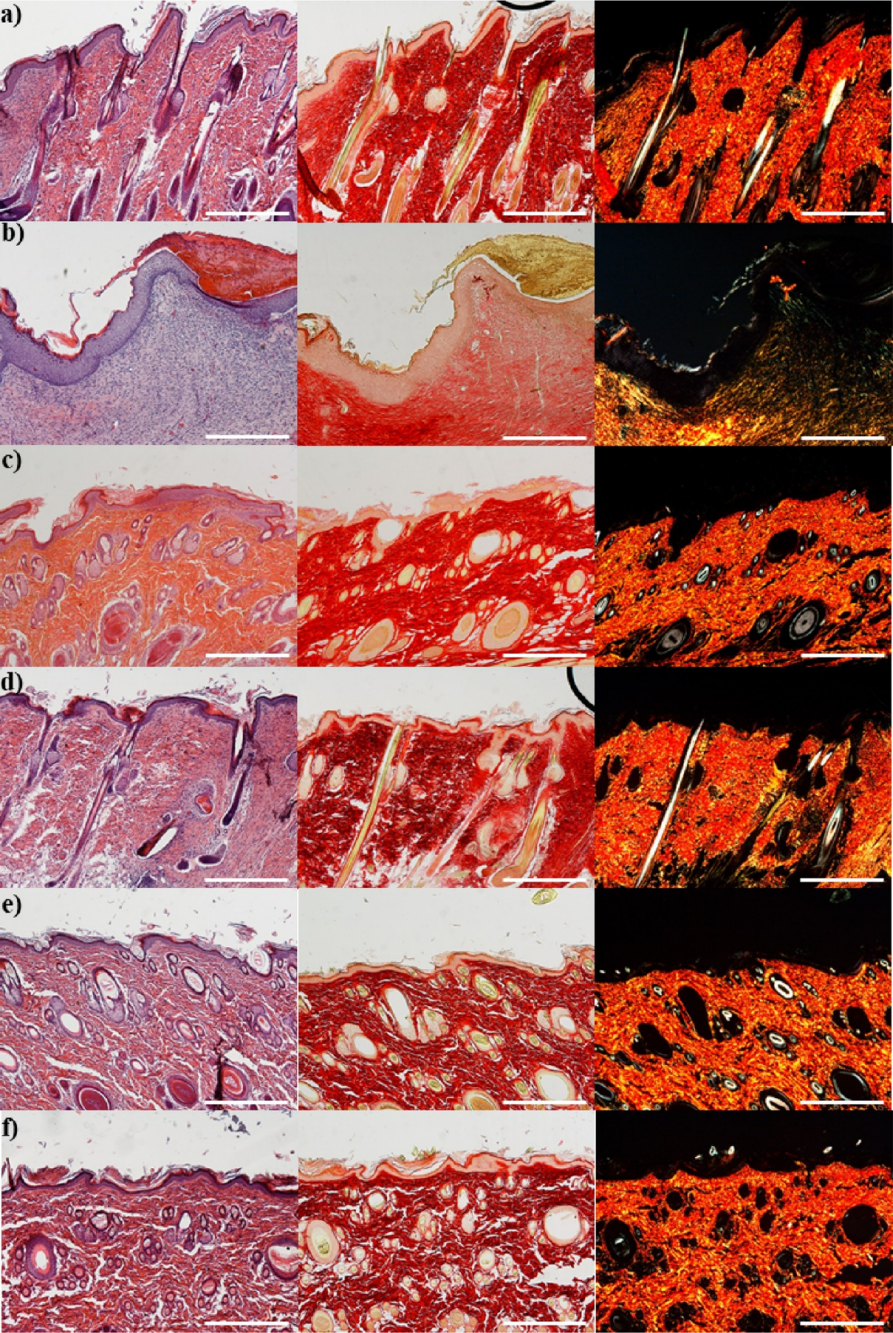
H&E and PSR sections of the (a) intact skin, (b) wound treated with saline solution, (c) subcutaneous implant of T12 scaffold, (d) subcutaneous implant of T12-CS scaffold, (e) subcutaneous implant of T12-CeO_2_ scaffold, and (f) subcutaneous implant of T12-CS-CeO_2_ scaffold. Original magnification: 5×. Each micrograph frame has a width of 1780 μm (scale bar: 500 μm).

The in vivo safety of the scaffolds was investigated in a murine excisional model; biopsies of intact skin (Figure 7a) and of the wound treated with saline solution (Figure 7b) were also taken as positive control.

After 18 days of treatment, completely regenerated epithelium is assessable in every sample. In particular, in the subcutaneous implant of all scaffolds, the wound area is hardly recognizable from the adjacent control skin, and collagen fibers are also remodeled in an orderly pattern. Subcutaneous T12-CS (Figure 7d) shows an epidermal layer fully restored in multiple layers of cells and with a fair degree of keratinization. Skin appendages such as hair follicles and glands are identical in number and disposition to those of intact skin. Subcutaneous T12, T12-CeO_2_, and T12-CS-CeO_2_ samples (Figure 7c,e,f, respectively) show well-formed keratinized squamous epithelium with normal collagen in the underlying dermis. Sebaceous glands and hair follicles are completely reformed, and hairs are about to erupt. No sign of inflammatory process is recognizable, with no leukocyte recruitment or foreign body response. In fact, Li et al. reported that CeO_2_ nanoparticles possess anti-inflammatory properties because they are able to regulate cytokine production from macrophages, protecting the surrounding tissues from the acute response. It is reasonable to hypothesize that CeO_2_ incorporation into the scaffolds could regulate the biological response of macrophages and mitigate host inflammatory response.^58,59^

An important issue could involve the possible accumulation of CeO_2_ nanoparticles in the body. It is already known that the liver is the major collector site after CeO_2_ administration.^60^ However, liver toxicity of CeO_2_ nanoparticles in healthy rodents by different administration routes has been extensively evaluated in the literature, and it was found to appear when higher doses are used, such as tenths or hundredths of mg of CeO_2_ per kg of animal. On the contrary, no toxic effects are usually observed in doses of few tenths of mg per kg of animal body weight, which is the case of the scaffolds developed in the present study. Hijaz et al. evaluated CeO_2_ nanoparticles conjugated with folic acid as a therapeutic agent in ovarian cancer, observing that an intraperitoneal injection of 0.1 mg kg^–1^ twice a week for 4 weeks in nude mice was not associated with histological alterations of the liver or alterations in the plasma biochemical measurements of liver function.^61^ Moreover, an implantation study showed that CeO_2_ did not show systemic toxicity or in vivo micronucleus induction in bone marrow.^62^ Furthermore, in vivo studies also present different shreds of evidence that CeO_2_ nanoparticles possess protective effects in liver disease, usually related to the use of lower doses ranging from 0.1 to 0.5 mg per kg^–1^.^60^

## 4 Conclusions

Nanofibrous scaffolds based on TPU and enriched with CeO_2_ and CS were efficiently manufactured. It was possible to develop fibers with an aligned shape able to imitate the tendon fascicles. The CeO_2_ was successfully embedded into the fibrous matrix, resulting in an increase of the scaffolds’ mechanical properties, especially when scaffolds were aligned, reaching values comparable to the ones of the native tendons. The weight loss test suggested a degradation in prolonged times. In particular, the scaffolds maintained their morphology and also remarkable mechanical properties after 12 weeks of degradation, which should guarantee an adequate support against the mechanical stresses during the entire tissue repair process. The scaffolds promoted the cell adhesion and proliferation, in particular when in aligned conformation. Moreover, the CS led to an increase in the TEN-1 adhesion and proliferation, and it seemed also to have a synergic effect with the CeO_2_ onto the cell growth. Finally, the systems in vivo did not cause any inflammatory effect: neither leukocyte recruitment nor foreign body response was observed.

The TPU’s great versatility and wide range of mechanical properties represent a great potential, and its application has been investigated in a wide range of fields in tissue engineering applications, from bone^63,64^ to vascular regeneration.^65^ However, the research is still poor in its application in tendon regeneration, where materials such as poly-l-lactic acid (PLLA),^66^ polyglycolic acid (PGA),^67^ and poly-dl-lactic-co-glycolic acid (PLGA)^68^ are the most studied. Nevertheless, these often fail in combining the appropriate mechanical properties and degradation rate, limiting their in vivo applicability.^69^ Moreover, a common issue with these more common materials is their poor physiological activities, such as the selective cell adhesion.^69,70^ The present study demonstrated that the developed scaffold could overcome these problems as a result of the combination of TPU, CeO_2_, and CS.

In conclusion, scaffolds based on TPU and doped with CS and CeO_2_ represent an interesting tool to enhance the tendon tissue regeneration and support the mechanical stresses. Further investigation on the scaffolds’ efficacy in vivo will evaluate their capability of enhancing the tendon ECM restoration, eventually accelerating their translation to the clinic.

## Supporting Information Available

Characterization of the polymeric blends (surface tension, consistency, and conductivity); EDX spectra of the electrospun scaffolds; SAXS patterns of the scaffolds at equilibrium, after elongation, and during dehydration; and equatorial and meridional intensity profiles (PDF)
