# Supplementary material for: Cerium Oxide and Chondroitin Sulfate Doped Polyurethane Scaffold to Bridge Tendons
Source: ACS Appl Mater Interfaces. 2023 May 23;15(22):26510–24. doi: 10.1021/acsami.3c06144 (PMC10251353; doi:10.1021/acsami.3c06144)
Supplement: Supplementary file 1 — am3c06144_si_001.pdf [file am3c06144_si_001.pdf]

SUPPLEMENTARY INFORMATION

Cerium oxide and chondroitin sulphate doped Polyurethane scaffold to bridge tendons

*Eleonora Bianchi[1], Marco Ruggeri[1], Barbara Vigani[1], Elena Del Favero[2], Caterina Ricci[2], Cinzia Boselli[1], Antonia Icaro Cornaglia[3], César Viseras[4], Silvia Rossi[1], Giuseppina Sandri[1]**

[1]Department of Drug Sciences, University of Pavia, Viale Taramelli 12, 27100 Pavia, Italy

[2]Department of Medical Biotechnology and Translational Medicine, University of Milan, LITA Viale Fratelli Cervi 93, 20090 Segrate, Italy

[3]Department of Public Health, Experimental and Forensic Medicine, University of Pavia, via Forlanini 2, 27100 Pavia, Italy

[4]Department of Pharmacy and Pharmaceutical Technology, Faculty of Pharmacy, University of Granada, Campus of Cartuja, 18071 Granada, Spain

*corresponding author's e-mail: g.sandri@unipv.it

1. EXPERIMENTAL SECTION

1.1 Characterization of Polymeric Blends. The measurement of consistency was conducted using the Texture Analyzer TA.XT plus (ENCO, Spinea, I), equipped with a cylindrical Perspex probe with the diameter of 20 mm (P/20P, batch No. 11434) at room temperature. To evaluate the solution consistency, it was evaluated the sinking force of the probe in the polymeric solution, that is influenced by the material characteristics, the temperature and the force applied on the probe.

For the surface tension measurements an automatic tensiometer was used (DY-300 Kyowa) and it was equipped with a platinum plate of 2.5cm x 1cm. The analysis conducted were time based, the instrument registered a tension value every 3 seconds for a total time of 300 seconds.

The conductivity was evaluated using a portable conductivity meter FiveGo F3 - Mettler Toledo. The instrument was calibrated before starting the analysis using two solutions with a known conductivity of 1413 μS/cm and 1288 μS/cm, then measurements were conducted at room temperature.

Each measurement was at least duplicated.

1.2 Scaffolds Chemico-physical characterization. An EDX analysis was performed by means of SEM analysis (Tescan, Mira3XMU, graphite sputtering, Brno, Czech Republich).

An analysis of the morphology during stimulation to mechanical stresses was performed by SAXS. The samples were cut into rectangular strips of about 1 by 6 cm and mounted directly on the x-ray beamline, as shown in Figure S1.

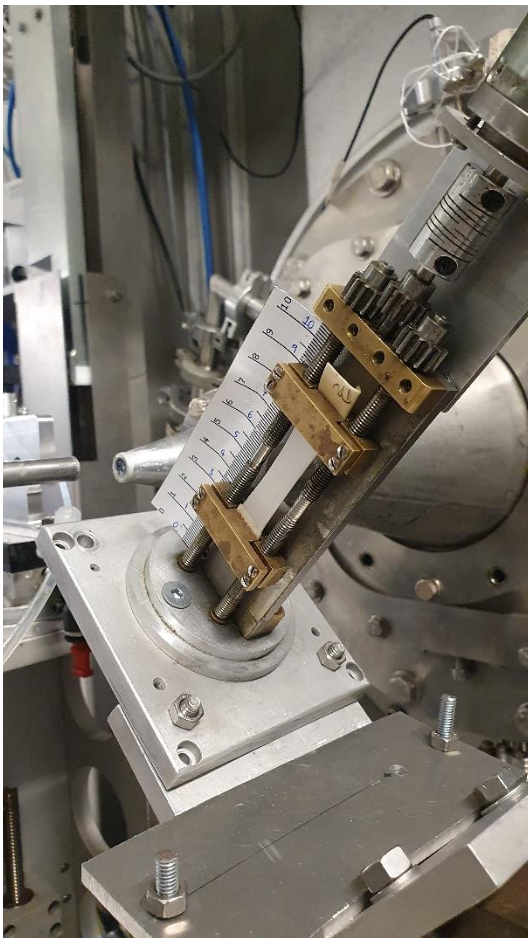

Figure S1. Sample holder for the analysis of the morphology during stimulation to mechanical stresses performed by SAXS.

2. RESULTS AND DISCUSSION

2.1 Characterization of Polymeric Blends. Table S1 reports the values of surface tension, consistency and conductivity of the undoped blends (T6, T9, and T12), and also of the blends loaded with CS and CeO_2. All the blends were characterized by similar values of surface tension, but their consistency and their conductivity were directly related to the polymer concentration, leading to electrospinning process enhancement. The loading of CS and CeO_2 did not lead to significant differences in the blends properties.

Table S1. Surface tension, consistency, and conductivity values of the polymeric blends (mean values ± s.d.; n = 3); ANOVA one-way; Scheffé test ($p < 0.05$): (surface tension) n.d.; (consistency) T6 vs T9, T12; T12-CS-CeO_2 vs T12, T12-CS, T62-CeO_2 (conductivity) T6 vs T9, T12; T9 vs T12; T12 vs T12-CS, T12-CeO_2, T12-CS-CeO_2

Blend	Surface tension (mN/m)	Consistency (mN)	Conductivity (μS/cm)
**T6**	32.42 ± 0.65	11.21 ± 2.02	1551 ± 4.36
**T9**	33.84 ± 0.11	14.01 ±1.48	1868 ± 6.24
**T12**	33.98 ± 0.47	14.75 ± 0.94	2061.5 ± 92.63
**T12-CS**	34.32 ± 0.20	14.41 ± 0.18	2219 ± 55.43
**T12-CeO_2**	34.53 ± 0.19	14.75 ± 0.94	2304.5 ± 33.23
**T12-CS-CeO_2**	34.05 ± 0.12	16.83 ± 0.07	2332 ± 57.37

2.2 Scaffolds Chemico-physical characterization. Figure S2 reports the EDX study performed on the T12, T12-CS, T12-CeO_2 and T12-CS-CeO_2 scaffolds.

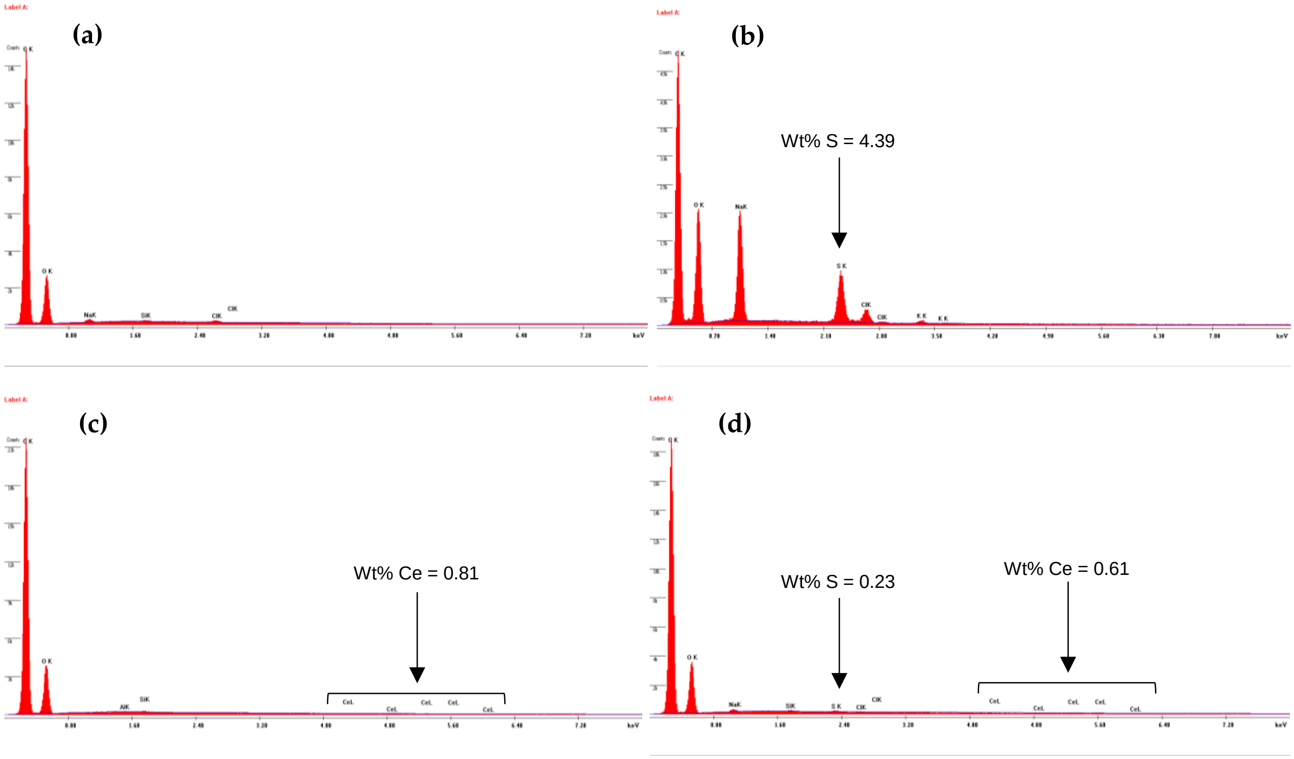

Figure S2. EDX spectra of a) T12, b) T12-CS, c) T12-CeO_2, and d) T12-CS-CeO_2 scaffolds

The EDX confirmed the presence of CeO_2 and CS in the TPU matrix as the T12-CS spectrum showed presence of sulphur (wt% 4.39), the T12-CeO_2 spectrum showed presence of cerium (wt% 0.81), and the T12-CS-CeO_2 spectrum showed presence of both sulphur and cerium (wt% 0.23, 0.61 respectively) in respect to the carbon and oxygen alone of the T12 spectrum.

2.3 Scaffolds structural characterization. Figure S3 shows the intensity 2D patterns of random R T12-CS scaffolds at 200% elongation during dehydration. The scattered intensity increase with dehydration is due to the higher contrast of fibers with respect to the air instead of water. The patterns become more asymmetric upon dehydration, revealing a different preferential elongation of the structure as a function of water content.

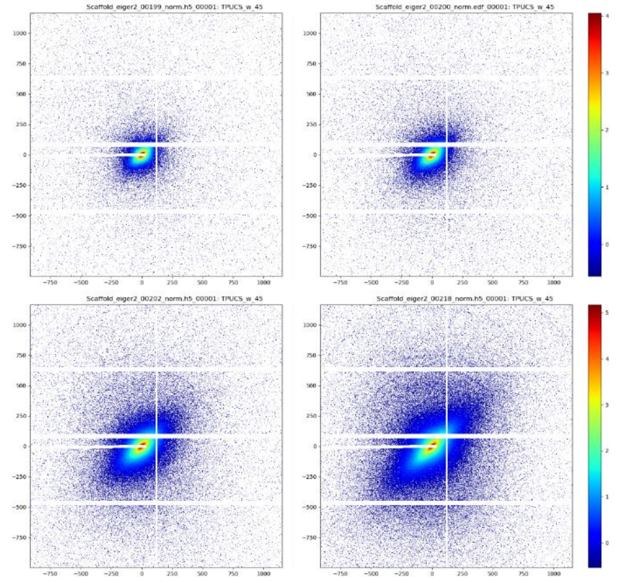

Figure S3. SAXS patterns of random R T12-CS scaffolds at 200% elongation during dehydration.
